# Usefulness of presepsin and procalcitonin levels in the diagnosis of sepsis in patients with acute kidney injury

**DOI:** 10.1186/cc13403

**Published:** 2014-03-17

**Authors:** Y Nakamura, H Ishikura, R Ichiki, K Hoshino, M Mizunuma, J Tanaka, A Murai

**Affiliations:** 1 Fukuoka University, Fukuoka, Japan

## Introduction

The presepsin (PSEP) and procalcitonin (PCT) levels are useful biomarkers for differentiating between sepsis and non- infectious systemic inflammatory response syndrome (SIRS). The PSEP and PCT levels have been reported to be abnormally high in patients with chronic renal failure. However, there have been no significant investigations regarding the relationships between these biomarkers and the presence of acute kidney injury (AKI) in the diagnosis of sepsis. The purpose of this study was to clarify the diagnostic accuracy of PSEP and PCT levels in patients with AKI.

## Methods

This study was conducted as a single-center retrospective study. Blood samples were collected from patients immediately after admission to the Department of Emergency and Critical Care Medicine, Fukuoka University Hospital between June 2010 and August 2013. We enrolled 629 patients in whom both the PSEP and PCT levels were measured on admission. We classified the patients into two groups according to the RIFLE criteria (Risk, Injury, Failure, Loss of kidney function and End-stage kidney disease: Loss and ESKD): the AKI group and the non-AKI group. The patients in the AKI group were further classified into the sepsis group and the nonsepsis group according to each stage of AKI. We subsequently investigated the diagnostic accuracy of the PSEP and PCT levels for detecting sepsis in these groups.

## Results

We evaluated 254 patients with AKI and 375 patients without AKI. The AKI group included 103 patients who met the Risk criteria, 65 who met the Injury criteria, 66 who met the Failure criteria and 18 who met the Loss and ESKD criteria. The mean PSEP and PCT levels were significantly higher in the sepsis group than in the nonsepsis group among the non-AKI patients and those meeting the Risk, Injury and Failure criteria (*P *< 0.01). The diagnostic accuracy of the PSEP and PCT levels for detecting sepsis was determined according to a ROC analysis; the area under the curve (AUC) for the PSEP and PCT levels was 0.883 and 0.870, respectively, in the non-AKI group. In addition, the AUC values for the PSEP and PCT levels in the Risk, Injury and Failure groups were 0.843 and 0.843, 0.818 and 0.922 and 0.669 and 0.804, respectively. In the Failure group, the AUC for the PSEP and PCT levels was 0.828 and 0.852, respectively, after dividing the PSEP and PCT levels by the creatinine (Cr) level. The optimal cutoff values for the PSEP and PCT levels for diagnosing sepsis were 409 pg/ml/Cr (sensitivity: 66.0%, specificity: 91.7%) and 1.5 ng/ml/Cr (sensitivity: 63.6%, specificity: 95.8%), respectively.

## Conclusion

The diagnostic accuracy of PSEP and PCT levels for detecting sepsis decreased in the Failure group; however, the value of these parameters for diagnosing sepsis in patients meeting the Failure criteria increased after dividing them by the Cr level.

